# The inverse association between relative abundances of oleic acid and arachidonic acid: a case of distribution dependent regulation?

**DOI:** 10.1186/s12944-019-1067-7

**Published:** 2019-05-28

**Authors:** Arne Torbjørn Høstmark, Anna Haug

**Affiliations:** 1 University of Oslo, Norway, Institute of Health and Society, Section of Preventive Medicine and Epidemiology, Box 1130 Blindern, 0318 Oslo, Norway; 2 The Norwegian University of Life Sciences, Department of Animal and Aquacultural Sciences, Box 5003, 1432 Ås, Norway

**Keywords:** Oleic acid, Arachidonic acid, Chickens, Random numbers

## Abstract

**Background:**

Since oleic acid (OA, 18:1 c9) and arachidonic acid (AA, 20:4 n6) may have antagonistic actions, it is of interest to assess their relative abundances. We previously reported an inverse correlation between % OA and %AA. However, percentages of the same sum may be correlated without involving biology. We now investigate whether random numbers, generated within the true concentration distributions for OA and AA, may be correlated.

**Methods:**

We reanalysed data from a previous diet trial in chickens. Breast muscle was collected, and the concentration of fatty acids in muscle lipids was determined using gas chromatography. We computed R = S – OA – AA, where S is the sum of all fatty acids (g/kg) and R is concentration of all fatty acids, except OA and AA. From histograms we found physiological distributions of OA, AA and R. Then we generated random numbers for each of 3 variables (*n* = 163), within the distributions (g/kg) for OA (1–7), AA (0.25–0.39), and R (4–10). Next we made scatterplots of % OA vs. % AA, and studied how a narrowing or broadening of distributions might change the relationship.

**Results:**

Also with random numbers, generated within true concentration distributions for OA and AA, we found an inverse relationship between their percentages (r = − 0.356, *p* < 0.001; r = 163); however, the points were not close to the regression line. The %OA vs. %AA relationship changed appreciably in response to slightly altering concentration distributions of OA and AA, and a negative association could be changed to become positive.

**Conclusion:**

Using random numbers, generated within the biological distributions for OA, AA, and sum of the remaining fatty acids, we found an inverse relationship between “% OA” and “% AA”, but the scatterplot was poor compared with that obtained with real values. The association between relative abundances of random numbers of OA and AA was very sensitive to changes in distributions, and a negative association could be changed to become positive by slightly altering the distributions. Thus, the association between relative abundances of OA and AA could be partly caused by the particular distribution of the fatty acid concentration: a Distribution Dependent Correlation.

## Background

We previously reported an inverse relationship between percentages of oleic acid (OA, 18:1 n9) and arachidonic acid (AA, 20:4 n6) in breast muscle lipids from chickens [1], in human serum phospholipids [2], and serum total lipids in the rat [3], suggesting that the OA vs. AA relationship might be a general one.

The bivariate association between relative abundances of OA and AA could be of biological significance since this couple of fatty acids seems to have opposing actions. It is widely accepted that oleic acid and foods rich in oleic acid such as olive oil may have beneficial health effects, such as improved insulin sensitivity, endothelium-dependent flow-mediated vasodilatation [4], lowering of LDL cholesterol [5, 6] and an increase in HDL cholesterol [7], reduced blood pressure [8], as well as anti-carcinogenic and anti-inflammatory effects [9–11]. If lipids in LDL are enriched in oleic acid, the particles will be less liable to be oxidized [12, 13]. Thus, many of the effects of oleic acid may serve to reduce the risk of cardiovascular diseases.

When considering the beneficial health effects of oils rich in oleic acid, we previously suggested [1–3] that many of the positive effects would be anticipated if the fatty acid works to counteract effects of arachidonic acid (AA, 20:4 n6). This fatty acid is formed in the body from linoleic acid (LA, 18:2 n6), a major constituent in many plant oils, and is converted by cyclooxygenase and lipoxygenase into various eicosanoids, i.e. prostacyclines, thromboxanes and leukotrienes [14]. AA derived thromboxane A_2_ (TXA_2_) and leukotriene B_4_ (LTB_4_) have strong proinflammatory and prothrombotic properties [15, 16]. Furthermore, endocannabinoides, which are derived from arachidonic acid, may have a role in adiposity and inflammation [17].

An interaction between oleic acid and arachidonic acid was suggested several years ago in the rat [18]. More recently, Cicero et al. [12] showed in human subjects that supplementation with a high dose of olive oil for 3 weeks resulted in an increase in LDL oleic acid and a decrease in linoleic and arachidonic acid. Also in chicken breast muscle a negative association between OA and AA was observed [19].

It seems that the Delta-9 desaturases are of considerable physiological significance. Thus, regulation of the amount of monounsaturated fatty acids has the potential to affect a variety of key physiological variables, such as insulin sensitivity, metabolic rate, adiposity, atherosclerosis, cancer and obesity [20, 21].

We previously suggested that feedback regulation between the synthesis of OA and AA could serve to partly explain the inverse relationship [1–3]; also, α-linolenic acid (ALA, 20:3 n3), and possibly other fatty acids, was suggested to be involved in the regulation. ALA is the precursor of the endogenous synthesis of EPA, and known to have many health effects [22]. Indeed, we observed in chicken breast muscle lipids that ALA appeared to stimulate formation of OA, and inhibit AA synthesis [1].

One mechanism by which OA could counteract those of AA is to reduce the relative abundance of AA in serum and tissues. Conceivably, increased supply of oleic acid might reduce that of AA by pure mass action. Inverse regulation could also be effected through more specific metabolic feedback regulation. For example, inhibition by AA of Delta-9 desaturase should lower percentage OA, and previous studies suggest that this latter mechanism might take place [20].

There is, however, a methodological concern when correlating percentages of for example OA and AA, since the same sum appears in the denominator when calculating the percentages. Thus, the relative abundances of OA and AA could be correlated without having any biological explanation. However, if so it is not a priori apparent whether the correlation will be positive or negative, and to what extent the association may be attributed to the concentration distribution and/or to specific biological regulatory mechanisms. In an attempt to shed some light on these questions we have reinvestigated our previously reported inverse relationship between percentages of OA and AA [1], and extended the study by replacing the observed concentrations for OA and AA with random numbers, generated within the real distributions. By this approach we anticipated to circumvent biological feedback mechanisms. Similarities and differences between results obtained with random and real numbers were visualized by scatterplots. As expected, preliminary analyses showed significant correlations also with random numbers. It then occurred to us that the inverse relationship between relative abundances of OA and AA might possibly be - at least partly - caused by the particular *distribution* of OA, AA, and sum of the remaining fatty acids (R). If so, then a change in distributions should disturb the %OA vs. %AA relationship. We accordingly extended the study to include analyses on how variations in the distributions of OA, AA, and R might influence the association between percentages of OA and AA.

## Methods

The present study is a spin-off of a previously published diet trial [1], in which groups of chickens were fed different types of diet; from this trial we reported an inverse correlation between % OA and %AA. However, percentages of the same sum may be correlated without involving biology. The aim of the present study was to clarify to what extent also random numbers, generated within the true concentration distributions for OA and AA, may be correlated. This work was carried out at The Norwegian University of Life Sciences (diet trial, AH), and at The University of Oslo, Norway (random number analyses, ATH).

### Chickens and diet

We refer to our previous article [1] for details concerning the diet trial. In brief, from day 1 to 29 one day old Ross 308 broiler chickens from Samvirkekylling (Norway) were fed wheat based diet with about 20% linoleic acid (18,2 n6) and 14% α-linolenic acid (18,3 n3) of the total fatty acid methyl esters. The animals had free access to feed and water. They were kept group wise in mesh floored battery cages from day 1 to day 12, and then placed in separate metabolism cages from day 12 until day 29. The birds were inspected twice daily by qualified handlers, and every other day by a veterinarian throughout the trial period. The feeds were all containing 8% supplement fat, of which 2.4% was linseed oil. At day 29, the animals were stunned by a sharp blow to the head and killed by exsanguination. Samples from the breast muscle were frozen at − 20 °C for fatty acid analyses as described by O’Fallon et al. [23]; the fatty acid composition of total lipids of breast muscle and feed was determined by gas chromatography. Fatty acid content is presented as g fatty acid/kg tissue (wet weight), and as weight percentage of the measured fatty acids.

### Statistical analysis

We first reanalysed our previously reported [1] association between %OA and %AA (scatterplot, see Fig. 2). We next computed S, the sum (g/kg we weight) of all fatty acids, and R, the remaining sum when omitting oleic acid (OA, 18:1 n9) and arachidonic acid (AA, 20:4 n6).

Thus, R = S - OA-AA. To determine distributions of OA, AA, and R, we made histograms. For analyses with random numbers, we generated uniformly distributed random numbers with the physiological distributions for OA, AA, and R. Since the diet trial had 163 chickens, for each of the variables OA, AA, and R we generated 163 random numbers. The physiological distributions (found from histograms) were: for OA 1–7, for AA 0.25–0.39, and for R 4–10. Percentages of the variables were computed: %OA = (OA/S)*100; %AA = (AA/S)*100; %R = (R/S)*100. We made histograms to illustrate the distributions of percentage values of OA, AA, and R. Minimum and maximum values of the percentages were also controlled with manual calculation. Dependency between percentages is shown by the equation %OA + %AA + %R = 100. Note that we use OA and AA also with random numbers to keep in mind that the aim of our analyses was to mimic results with real values of this couple of fatty acids, but upper case letters was used (RANDOM) in the figure texts to clarify. Using random numbers, generated within the physiological distributions for OA, AA, and R, we made scatterplots for (RANDOM number) %OA vs. %AA. We additionally used random numbers for OA and AA only, while keeping the physiological R values; the outcome was qualitatively as with the random R numbers (results not shown). Finally, we studied how alterations in the distributions for OA, AA, and R might change the relationship between %OA and %AA. Since there are infinite numbers of ways to change the distributions, we limited our analyses to narrowing or broadening of the physiological distributions, and to change distribution of only one of the variables each time. For each analysis, we made several repeats with new sets of random numbers; the outcome of the repeats was always the same, but the correlation coefficients (Pearson) varied slightly. Results are mainly presented as scatterplots. SPSS 25.0 was used for the analyses, and for making figures. The significance level was set at *p* < 0.05.

## Results

### Concentration distributions for OA, AA, and sum of the remaining fatty acids (R)

**Fig. 1:**
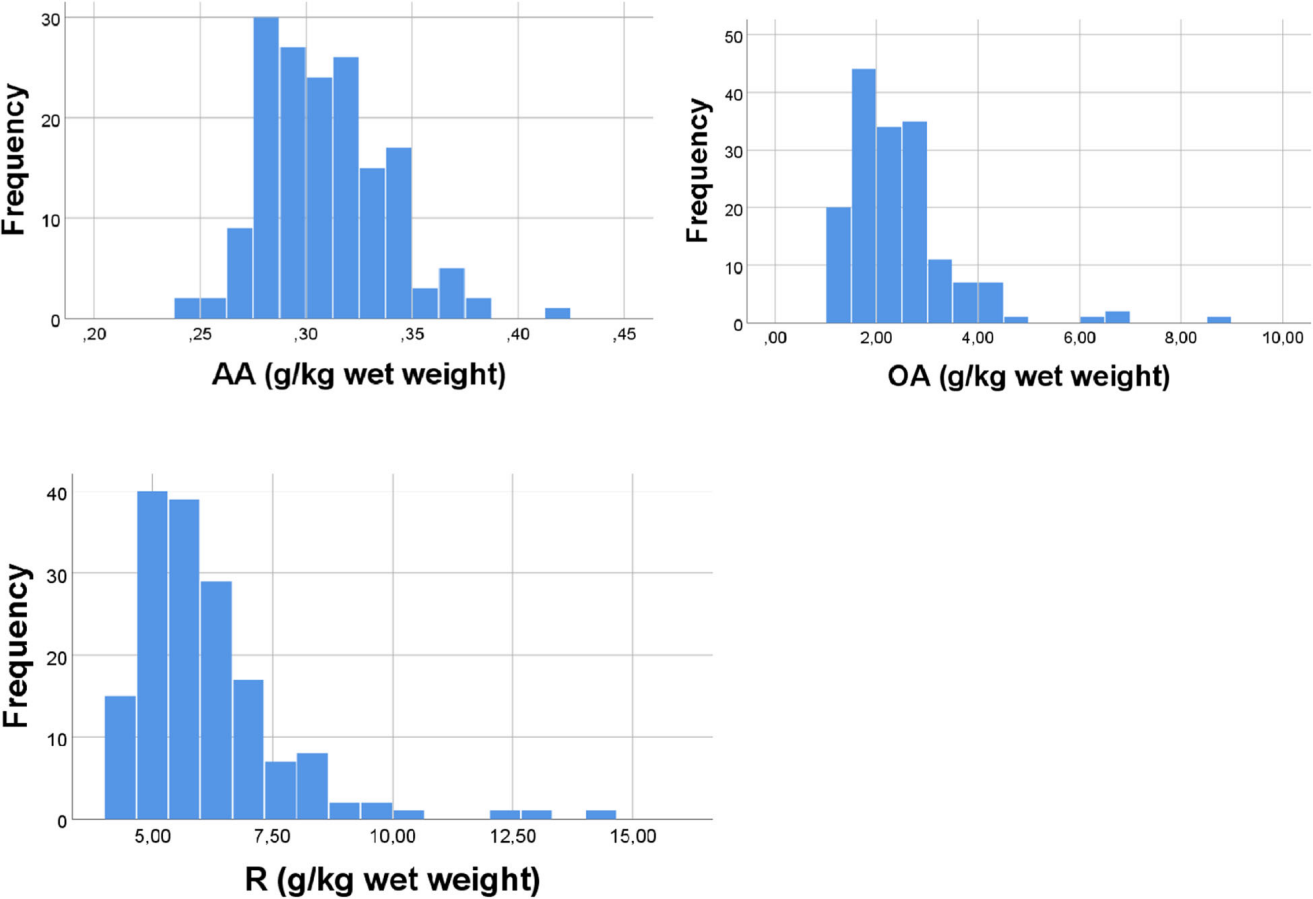
Physiological distribution of fatty acids Distribution of AA (20,4 n6, top left), OA (18,1 n9, top right), and sum of the remaining fatty acids (R) in breast muscle lipids of chickens (*n* = 163)

There was a considerable variation in mean amounts of the various fatty acids, ranging from 0.01 to 2.44 g/kg. Mean sum of all fatty acids was 8.86 ± 0.21 (*n* = 163) g/kg. Mean percentage of OA and AA was 26.7 and 3.7%, respectively. Concentration distributions for AA, OA, and R are shown in Fig. 1. To mimic the real distributions shown in Fig. 1, when generating the 163 random numbers for each 3 scale variables (vide infra) we used the following distributions: 0.25–0.39 for AA, 1–7 for OA, and 4–10 for R (sum of the remaining fatty acids).

### Association between percentages of OA and AA: scatterplots with real values and random numbers

**Fig. 2:**
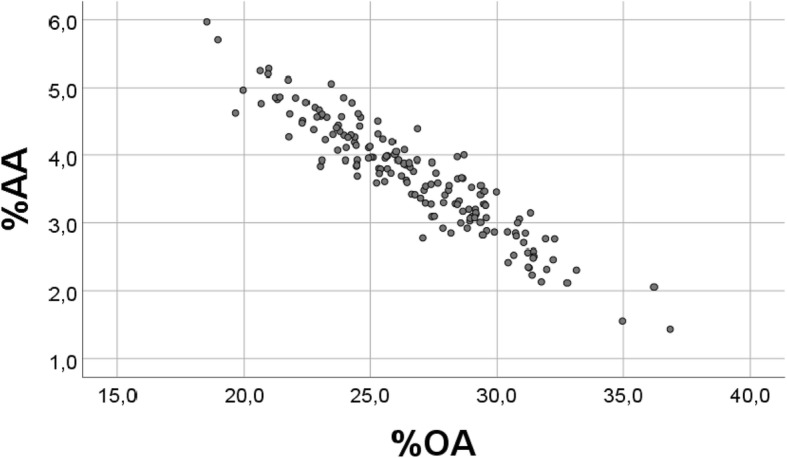
Relationship between percentages of OA and AA in chicken breast muscle lipids. Scatterplot of %OA vs. %AA, with real values, obtained in a diet trial in 163 chickens, see Methods (r = − 0.935, *p* < 0.001)

**Fig. 3:**
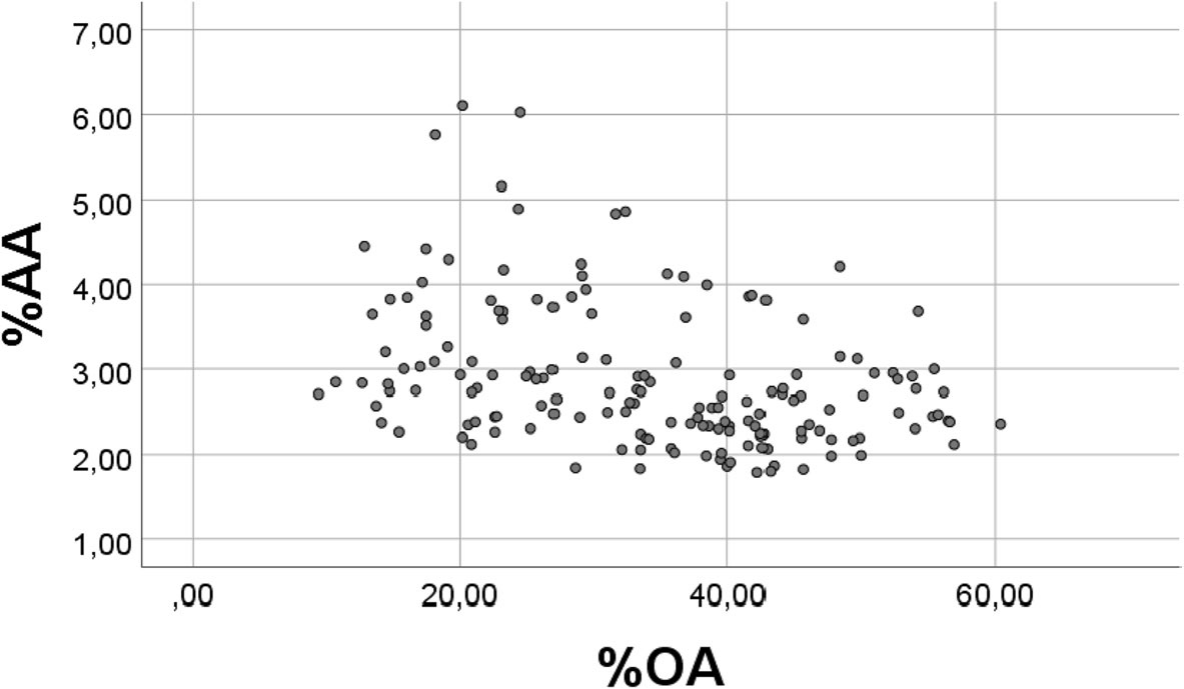
Relationship between RANDOM number “%OA” and “%AA”. For each of the variables, real values were replaced with 163 RANDOM numbers, generated in the physiological distributions; for OA (1–7), for AA (0.25–0.39), and for R (4–10). This figure should be compared with Fig. 2, where real values were used (r = − 0.356 (p < 0.001)

There was a strong inverse association between percentages of OA and AA (r = − 0.935, *p* < 0.001, *n* = 163, Fig. 2). However, also when using random numbers instead of the real values, but keeping the random numbers within physiological distributions, we found a significant inverse association (r = − 0.356, *p* < 0.001 (n = 163) between %“OA” and % “AA” (Fig. 3). Note that we keep using OA and AA also when presenting results with random numbers (vide infra), but write upper case RANDOM in the figure texts to clarify. In spite of high significance (p < 0.001) also with random numbers, Fig. 3 demonstrates a poor scatter as compared with that obtained with real values. When repeating the analysis with new sets of random numbers, the outcome was always the same, but with slightly differing values of the correlation coefficients.

Thus, for each of the variables, real values were replaced with 163 random numbers, generated with the physiological distributions for OA (1–7.00) and for AA (0.25–0.39). Fig. 3 should be compared with Fig. 2, where real values were used.

### How does the %OA vs. %AA association respond to variations in the distribution of OA, AA, and R?

We next investigated whether changes in the physiological distributions of OA, AA, and sum of the remaining fatty acids (R), might influence the %OA vs. %AA relationship. Since there are infinite many ways to change the distributions, and their degrees of overlap, we limit our analyses to mainly study effects of either narrowing or broadening the physiological distributions, and to change only one of the variables each time. Implicit in the changes of OA, AA, and R distributions is that also the total sum of fatty acids must change.

### Changing the distribution of OA

#### Narrowing the distribution

**Fig. 4:**
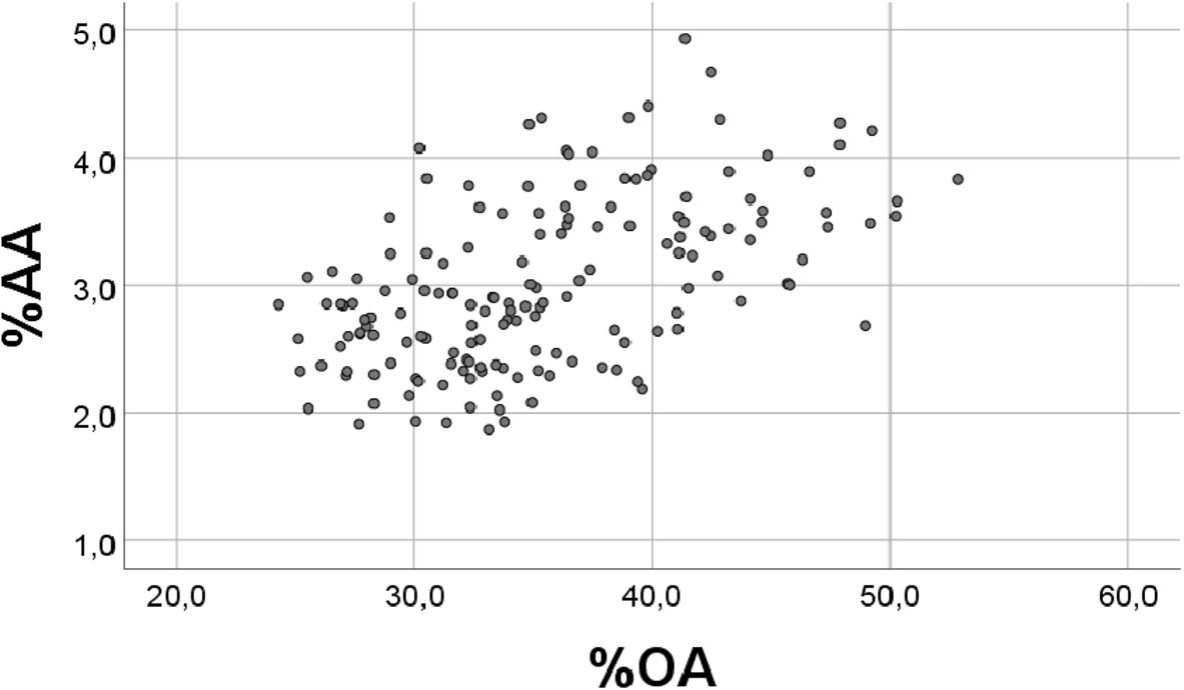
Relationship between percentages of “OA” and “AA”, obtained with a narrowing of the OA distribution. Real values were replaced with 163 RANDOM numbers, generated with the physiological distribution; for AA (0.25–0.39) and R = (4–10), but with an altered distribution of OA, i.e. 3–5 (instead of 1–7), see Methods (r = 0.528, p < 0.001)

The %OA vs. %AA relationship was very sensitive to changes in the distribution of OA. A small narrowing of the OA distribution to 2–7 instead of 1–7, made the negative %OA vs. %AA relationship become non-significant (r = − 0.005, *p* = 0.951). Increasing the narrowing of the OA distribution even more, to 3–5 (instead of 1–7), and keeping physiological distributions for AA (0.25–0.39) and R (4–10), had the effect that the negative %OA vs. %AA relationship was changed to become positive; r = 0.528, *p* < 0.001 (Fig. 4).

We then narrowed the OA distribution further, to 3–4, but keeping the “physiological” values for AA and R. The positive %OA vs. %AA association was improved (r = 0.694, *p* < 0.001, not illustrated). Thus, by a progressively narrowing the OA distribution we obtained that a negative association between percentages of OA and AA first changed to be non-significant, and then to be positive.

#### Broadening the distribution of OA

When broadening the OA distribution to 0.5–12 (instead of 1–7), and keeping distributions of AA and R, there was an improved negative association between %OA and %AA (r = − 0.591, *p* < 0.001, not illustrated).

### Changing the distribution of AA

#### Narrowing the distribution

Our analyses suggest that the inverse relationship between percentages of OA and AA is more resistant to changes in the distribution of AA than to alterations in the OA distribution. When narrowing the AA distribution to 0.28–0.30 (instead of 0.25–0.39), there was still a significant negative correlation between %OA and %AA (r = − 0.294, *p* < 0.001).

#### Broadening the distribution of AA

If broadening the AA distribution to 0.15–1.0 (instead of 0.25–0.39), we found a minor change of the negative correlation between percentages of OA and AA (r = − 0.231, *p* = 0.003).

### Changing the distribution of R

#### Narrowing the distribution

**Fig. 5:**
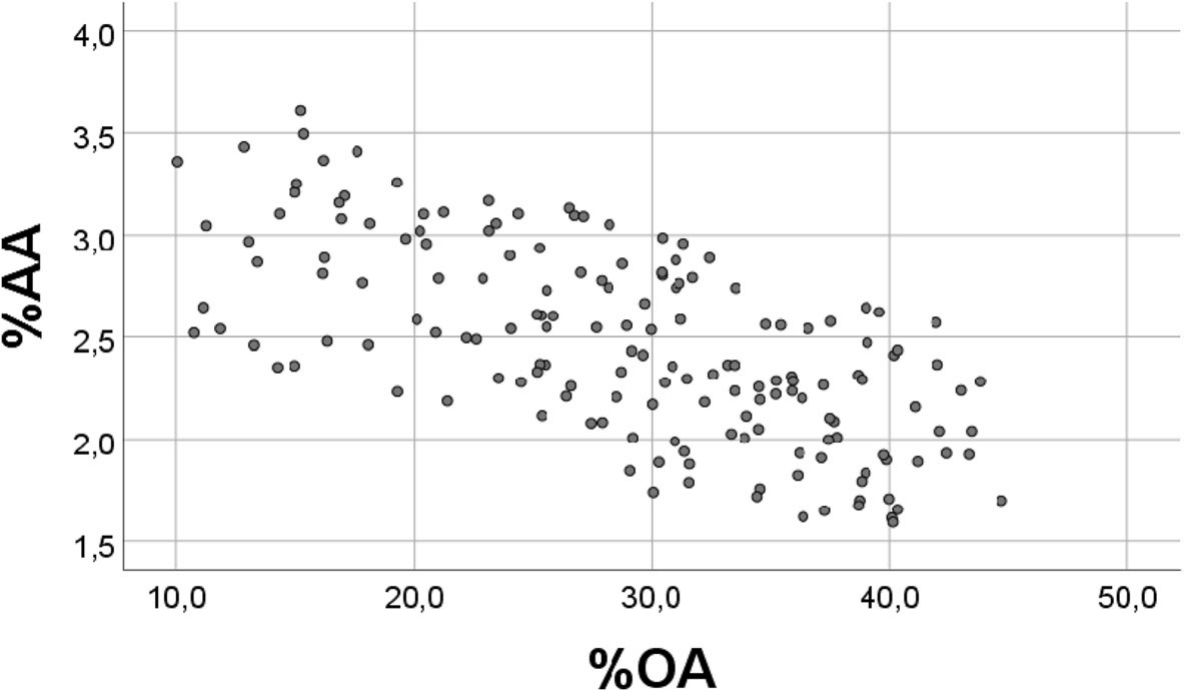
“%OA” vs. “%AA”, obtained with a narrowing of the distribution of R. Real values were replaced by 163 RANDOM numbers, generated in the physiological distributions for AA (0.25–0.39) and OA (1–7), but the distribution of R was changed to 8–10 (instead of 4–10), see Methods (r = − 0.669, p < 0.001)

By narrowing the distribution of R to 8–10 (instead of 4–10), and keeping the physiological distributions for OA and AA, we obtained an improved inverse association between %OA and %AA, r = − 0.669, p < 0.001 (Fig. 5).

#### Broadening the distribution

We first made a small broadening of the R distribution, from 4 to 15 (instead of 4–10) while keeping physiological ones for OA and AA. This alteration had the effect that there was no longer a significant association between %OA and %AA (r = 0.128, *p* = 0.104). To investigate the effect of a large broadening of R (far out of the physiological distribution), we made R run from 4 to 40, without altering distributions of OA or AA. By doing this, we obtain more of high values for the sum (S) of all “fatty acids”. Thereby, all numbers for AA and many OA values became small compared with R. We would accordingly expect that both %AA and %OA became reduced upon increasing S, and that both were negatively correlated with S. If so, we would expect that %OA and %AA were positively associated. Correlation analyses indicate that these assumptions were correct: %AA vs. S: r = − 0.852 (*p* < 0.001); %OA vs. S: r = − 0.707 (p < 0.001); %OA vs. %AA: r = 0.625 (p < 0.001). Thus, the inverse association between %OA and %AA can change from negative to positive also in response to solely altering the distribution of R (scatterplot not shown).

These analyses with 163 random numbers for each of three variables, OA, AA, and R, show that the distribution per se can determine whether relative abundances of OA and AA are significantly correlated or not, and whether they are negatively or positively associated. We therefore suggest the existence of a *Distribution Dependent Regulation* of the association between percentages of fatty acids.

### Suggested explanation of the results

The present results strongly suggest that the inverse relationship between percentages of scale variables with distributions like OA and AA can change appreciably, and even turn from being negative to become positive by slightly changing distributions. First we consider the eq.

OA + AA + R = S, where OA and AA represent random numbers, generated with physiological distributions for oleic and arachidonic acid, and R is the sum of the remaining fatty acids. Thus, the S distribution depends on distributions of OA, AA, and R. In our analyses, a new set of random numbers were generated for each of our 163 cases, and each of the variables. Thus, there are an infinite number of combinations of values for OA, AA, and R. There is, however, the important limitation that we used the physiological distributions, i.e. 0.25–0.39 for AA, 1–7 for OA, and 4–10 for R. Dependency between the percentages is shown by the equation %OA + %AA + %R = 100. To better understand how the extreme values for percentages are brought about, we did manual calculations to find the lowest and highest percentages for AA, OA and R (Table 1).

**Table 1:** Lowest and highest absolute values of AA, OA, and R (g/kg wet weight), and of their percentage values

Variable	Absolute distribution (g/kg)	Distribution of percentage values
AA	0.25 - 0.39	Lowest: 100* 0.25/(0.25 + 7 + 10) = 1.4
		Highest: 100* 0.39/(0.39 + 1 + 4) = 7.2
OA	1 - 7	Lowest: 100* 1/(1 + 0.39 + 10) = 8.8
		Highest: 100* 7/(7 + 0.25 + 4) = 62.2
R	4 - 10	Lowest: 100* 4/(4 + 7 + 0.39) = 35.1
		Highest: 100*10/(10 + 0.25 + 1) = 88.9

R is the sum of all fatty acids, except AA and OA

For example, the lowest percentage of AA is obtained with the lowest value of AA (0.25) combined with the highest values for OA (7) and R (10), giving the lowest AA percentage (1.4%, Table 1). The calculated minimum- and maximum percentages for all of the three variables are shown in Table 1.

#### Negative correlation between percentages

**Fig. 6:**
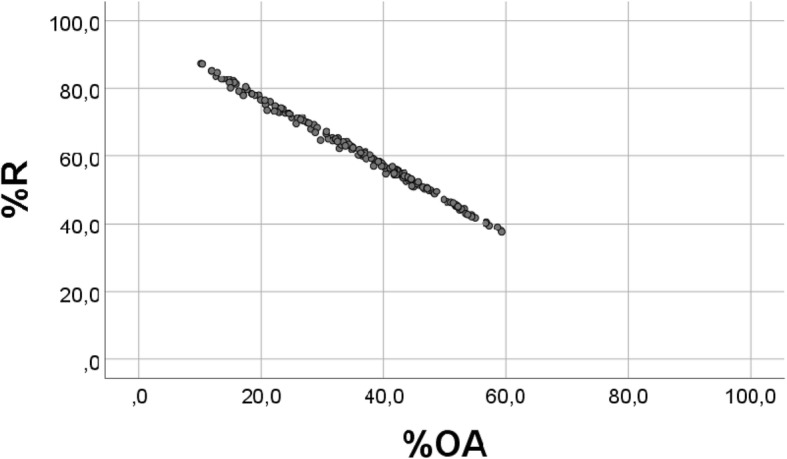
Scatterplot of RANDOM “%OA” vs. RANDOM “%R”. Real values were replaced with 163 RANDOM numbers, generated with physiological distributions, for OA (1–7), and R (4–10), see Methods (r = − 0.998, p < 0.001)

We rearrange the equation: %OA = − %R + (100 – %AA). Since AA is smaller than OA and R, %AA covers a smaller distribution than %OA and %R (Table 1). Thus, it seems justified to approximate the equation to: %OA = − %R + 100, showing a perfect inverse relationship between %OA and %R (Fig. 6). If we hypothetically extrapolate %OA to be zero, then %AA should approximate 100%. Conversely, extrapolating %R to zero would give %OA = 100, as also indicated in Fig. 6 (r = − 0.998, *p* < 0.001).

#### Positive correlation between percentages

We may consider 3 scale variables in general, A, B and C, giving %A + %B + %C = 100, i.e. % B = − % A + (100 - % C). If %C consists of high values (close to 100) and corresponding values of %B and %C are such that (100% - %C) > %A, then the equation will approach %B = %A, showing a linear positive association between %A and %B, That the requirement (100 - %C) > %A is indeed satisfied follows from this example: Suppose that %C could theoretically reach 99% (or any higher value), then the remaining percentage must be divided between %A and %B. Hence, %A will be positive. One example to approximate this situation is to let A and B both have the same distribution involving small numbers, for example from 0.10 to 0.15, and C a distribution involving higher numbers than A and B, e.g. from 1.0 to 10.0. A computer-check verified that, with these distributions, the requirements above were valid; i.e. the major %C-distribution was high (95 to 97%), and (100 - %C) > %A- (output not shown). Percentages of A and B correlated positively, r = 0.941, *p* < 0.001; equation of the regression line was (SE in parentheses) %B = 0.97 (0.02)*%A + 0.09 (0.07).

Thus, a positive association between %B and %A should be expected with very high %C values, making the expression (100 - %C) approach zero. However, unless A and B have the same distribution, it is inappropriate to write %B = %A, like Y = X. In the latter case, both the abscissa and the ordinate may have any value on the scale, and the Y vs. X graph would have slope = 1. In contrast to this, %A and %B – values are limited by the A and B ranges, respectively. A more appropriate equation would be: %B _(p - q)_ = − %A _(r - s)_ + (100 - %C _(t – u)_), where the subscript parentheses indicate ranges of A, B, and C. The slope of the %B vs. %A regression line will accordingly be determined by the distributions of A (%A) and B (%B). If A and B both have the same distribution, then the slope should be close to 1, as was observed in the above experiment. With different ranges for A and B, e.g. for A 0.20–0.40, for B 0.15–0.15, and for C 1–10, the equation of the regression line was: %B = 0.38 (0.01)* %A + 0.22 (0.10). We additionally did a manual calculation based upon minimum and maximum values of the A (%A) and B (%B) distributions. Using (max - min) of %B, divided by (max - min) of %A as a crude slope estimate, we found slope values to be 1.09, and 0.38, respectively, in the two latter examples above.

#### Suggested general rules for distribution dependent correlations

With reference to the considerations above and the equation %B = −%A + (100 - %C), there seems to be some general rules for *Distribution Dependent Correlations* between percentages of 3 positive scale variables: 1) the closer %C is to 100, the better is the positive association between percentages of A and B. Conversely, 2) the closer %C is to zero, the better is the negative association btw %A and %B. High (low) %C values are obtained with high (low)-number distribution of C, relative to the A (B) distribution. It follows that progressively increasing (decreasing) the C distribution towards high (low) values will improve (make poorer) a positive association between percentages of A and B, whereas the opposite outcome is expected for a negative correlation between %A and %B, in response to these distribution changes. Furthermore, we should expect a particular *Turning Point* [24] where a positive (negative) correlation between %A and %B turns to become negative (positive) in response to progressively altering distributions of C (and consequently of percentages of C, B, and A). These considerations seem to explain the observed alterations in scatterplots and correlation coefficients for %OA vs. %AA when altering distributions of OA, AA, and R. Below we briefly present some results to test the hypothesis, using cutoff values of %R quartiles to assess whether the %R distribution moves towards higher or lower value. We use the previous, special equation: %OA = −%AA + (100-%R). To better clarify the response, we will exemplify by using *large hypothetical alterations* in variabilities of the surrogate, random numbers for OA, AA, and R, however by changing one of the variables only in each experiment. First we let R go from 4 to 20 (keeping in mind the physiological R distribution 4–10). As expected, the negative correlation between percentages of “random number OA” and “AA”, had changed to become positive; r = 0.238, *p* = 0.002. Cutoff values for %R quartiles *before* broadening the R distribution were *52.9, 61.0, and 69.2%,* against *67.2, 75.4, and 83.3%*, respectively *after* broadening. Thus, the %R distribution had moved towards higher values, an effect *not* favoring a negative correlation between %OA and %AA, but rather a change towards a positive correlation. Thus, the *Turning Point* had been passed. We next narrowed R, to go from 4 to 5. The negative %OA vs. %AA correlation was greatly improved; r = − 0.849 (*p* < 0.001). A*fter* narrowing, the cutoff values for %R quartiles were *44.2, 50.6, and 61.3%,* respectively, i.e. the %R distribution had moved towards lower values, in favor of improving the negative correlation between %OA and %AA. High (low) values of %R should be achieved also by low (high) % OA (%AA). We accordingly narrowed OA to 1–2 (instead of 1–7). The %OA vs. %AA correlation had changed from negative to become positive; r = 0.638, *p* < 0.001. *After narrowing*, the cutoff values for %R quartiles were 75.4*, 79.4, and 82.8%.* Thus, the %R distribution had moved towards higher values, thereby favoring a positive %OA vs. %AA association. Conversely, a *broadening* of OA towards higher values, e.g. 1–17 (instead of 1–7) resulted in an improved negative %OA vs. %AA association, r = − 0.660, *p* < 0.001. A*fter broadening* OA, the cutoff values for %R quartiles were 34.8*, 43.8, and 58.3%,* respectively, against 52.9*, 61.0, and 69.2% before* broadening. This change in the %R distribution towards lower values should improve the %OA vs. %AA association, as was also verified by the correlation coefficient (and scatterplot, not presented). We next studied whether also large changes in AA might influence the %OA vs. %AA relationship, and first narrowed AA, i.e. 0.25–0.26. The %OA vs %AA correlation was; r = − 0.277, p < 0.001. A*fter AA- narrowing,* the cutoff values for %R quartiles were *53.3, 62.7, and 73.1%,* respectively, against 52.9*, 61.0, and 69.2% before narrowing AA*, i.e. there had been a minor change in the %R distribution towards higher values, and a minor change towards a poorer %OA vs. %AA correlation should be expected, as was also found. We finally studied the effect of *broadening* AA towards higher values, using the range 0.25–3.25. In this case; for %OA vs. %AA: r = − 0.443, p < 0.001. A*fter broadening* AA, the cutoff values for %R quartiles were 48.6*, 55.1, and 62.8%,* respectively, i.e. a change in the %R distribution favoring an improved negative %OA vs. %AA association, as was verified by the correlation coefficient (and scatterplot, not shown).

**Fig. 7:**
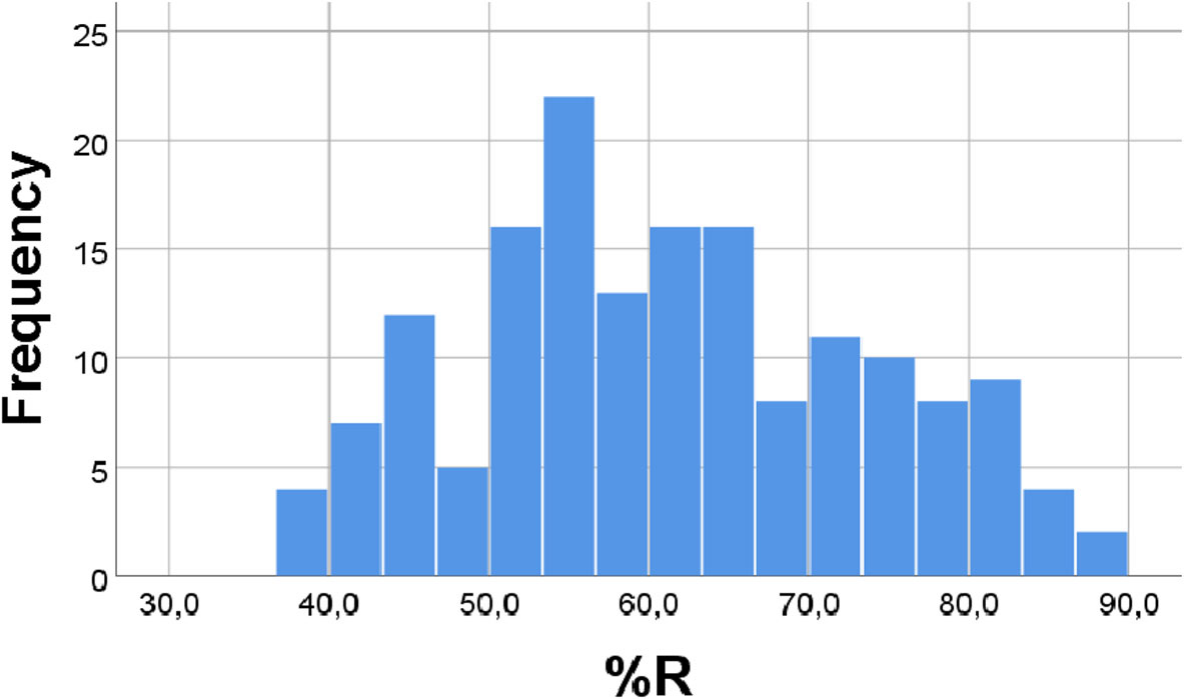
Histogram of RANDOM “% R”. Real values for OA and R were replaced with 163 RANDOM numbers, generated with the physiological distributions for R (4–10), and OA (1–7), see Methods

**Fig. 8:**
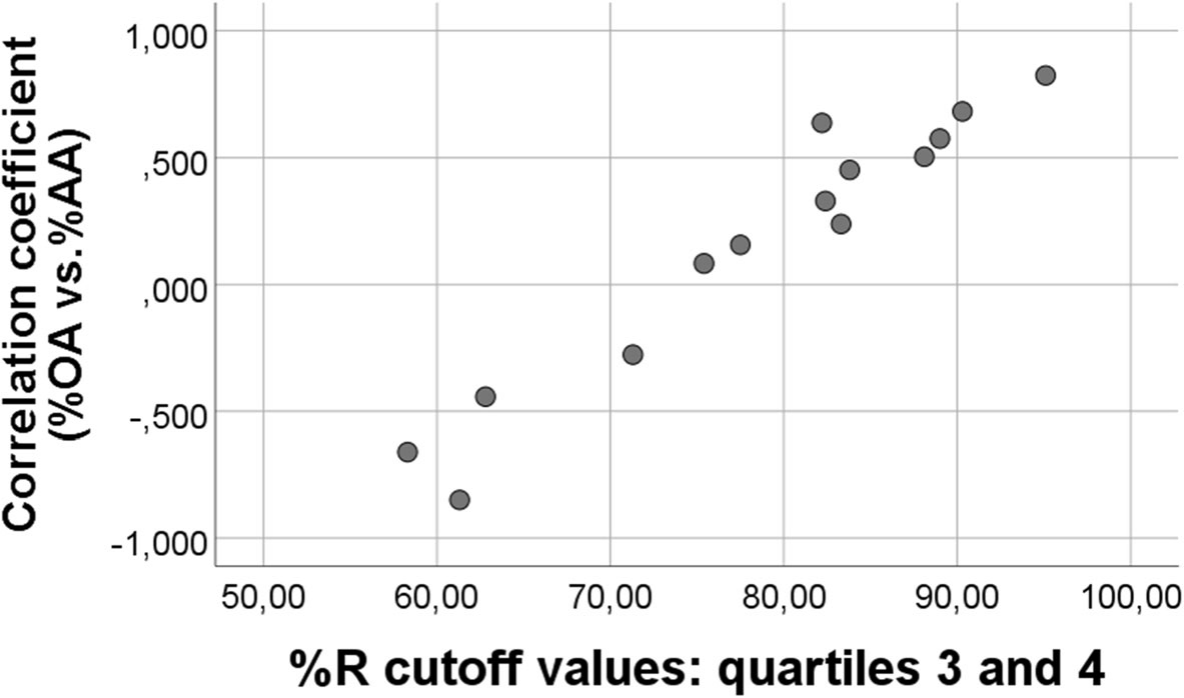
Relationship between cutoff values (between the 3rd and 4th quartile of the %R distribution) and corresponding r-values for the association between percentages of RANDOM number values for OA and AA. The figure refers to the equation %OA = −%AA + (100 -%R); see text for explanation. Each point on the scatterplot represent a particular situation where distribution of either R, OA, or AA have been altered in a computer experiment. Spearman’s rho for the association between values on the axes rho = 0.925, p < 0.001, *n* = 14

These examples illustrate that the equation %OA = −%AA + (100 - %R) seems to work well to explain the random number experiments shown in this work. Our analyses also seem to suggest that the physiological values of OA and AA are such that they do not clearly favor either a positive or a negative association between their percentages. Indeed, the particular distribution of % R (Fig. 7) seems to be somewhere in-between distributions favoring a negative or a positive OA vs. %AA association, making it hard to predict the correlation outcome without doing a computer analysis. Indeed, as predicted from the general equation above, by systematically varying distributions of the three variables, at a certain *Turning Point,* a negative (positive) correlation between percentages will change to become positive (negative), as discussed in more detail previously [24]. Anyhow, this reasoning serves to explain why even small changes in distributions may change the “%OA” vs. “%AA” association from being negative to become positive, as shown with random numbers in this work. However, the nice physiological, inverse association between percentages of OA and AA observed in the present diet trial in chickens is probably mainly attributed to other causes than the particular fatty acid distribution per se*.* The association between the 3rd and 4th quartile of the random number %R distribution and corresponding correlation coefficients for (random number) %OA vs. %AA is summarized in Fig. 8; some additional points have been calculated and added to the figure (obtained by increasing the R distribution; details omitted). This figure suggests that the positive (negative) association between “random number %OA and %AA” improves as the “%R” distribution moves towards higher (lower) values, as predicted above from the equation %OA = −%AA + (100 -%R). Figure 8 also shows that the *Turning Point* between positive and negative “%OA vs. %AA” correlation occurs when the cutoff between the 3rd and 4th quartile of the “%R” distribution is approximately 75%.

We did a series of additional random number analyses to study how % OA vs. %AA might change in response to changing the “physiological distributions” for each of the variables. In line with the reasoning above, we observed many times that even minor alterations in distributions can strongly influence, not only the size of the correlation coefficient and pattern of scatterplots, but also govern whether an association between relative abundances of OA and AA will be negative, positive, or non-existing. Also, in spite of poor values for the correlation coefficients and poor scatterplots, when repeating our analyses with new sets of random numbers generated within the same distributions, the outcome was always the same, but with slightly differing sizes of the correlation coefficients.

#### Are there distribution related negative correlations between %OA and %AA in previous studies?

We previously reported an inverse relationship between %OA and %AA also in rats (*n* = 36) and in 46 human subjects [2, 3]. However, replacing the measured values with random numbers having the same distribution did not give any significant relationship between the percentages of OA and AA. This finding apparently does not seem to support that distribution per se - in general - is a mechanism explaining the inverse %OA vs %AA association. However, the materials were quite different. In present work we analyzed fatty acids in breast muscle lipids of 163 chickens; in the rat study we measured fatty acids in total serum lipids of 36 male rats, and in the study of human subjects (11 men, 35 women): fatty acids in the phospholipid fraction of serum. For many fatty acids, there are appreciable differences between species/tissues in corresponding ranges and variabilities [25]. As explained above, to obtain a distribution related correlation, the crucial point is where on the scale the fatty acid concentrations are found, and their variabilities. It would appear that, in breast muscle lipids of chickens, distributions may favor a poor negative correlation between percentages of OA and AA. In contrast to this, the negative %OA vs. %AA association found in serum fatty acids of humans and rats does not appear to be partly explained by the fatty acid distribution per se.

We carried out some additional analyses to further examine whether distributions (range, variability) might cause a negative %OA vs. %AA relationship in serum lipids. In this regard we used published data [26] from young, healthy Canadians (Caucasians). Using the reported mean (SD) values to generate random numbers (*n* = 287) we did not find a significant inverse association between “%OA” and “%AA”. However, using the reported mean values, but applying variabilities found in our homogenous chicken population, we found a significant negative correlation between “%OA” and “%AA” (r = − 0.313, *p* < 0.001). Obviously, it is not justified to draw conclusions related to the association between percentages of OA and AA in human plasma total lipids based upon these calculations, since random numbers and hypothetical variabilities were used. Nevertheless, the calculations illustrate that percentages of “OA” and “AA” could be negatively associated as a consequence of distribution alone. Thus, for fatty acids in breast muscle lipids of chickens, it seems that the distribution per se may partly explain the inverse %OA vs. %AA correlation, but apparently not in our two previous studies, one in sera of human subjects, and another in sera of rats. In this context we may add that distribution alone does seem to completely explain the *positive* association observed between percentages of AA and EPA in breast muscle lipids of chickens [27, 28], a finding strongly suggesting that there might be a *Distribution Dependent Regulation* of the correlation between percentages of some fatty acids.

## Discussion

These analyses suggest that the distribution per se of three random scale variables can determine whether the association between their relative abundances is negative, positive, or not significant. In our examples we have used random numbers generated within the physiological distribution of OA and AA, and R (sum of the remaining fatty acids) found in breast muscle lipids of chickens. Our analyses suggest that there may partly be a *Distribution dependent correlation* of the association between relative abundances of OA and AA in this tissue. This conclusion raises the intriguing question of whether this type of regulation is of any biological interest, or just a correlation bias.

### Is our previously reported inverse relationship between %OA and %AA just a case of distribution dependent regulation?

We previously suggested that the inverse association between percentages of OA and AA, observed in serum phospholipids of young, healthy subjects [2], in total serum lipids of rats [3], and in breast muscle lipids of chickens [1] might be attributed to a direct feedback regulation between the synthesis of OA and AA, and also governed by ALA [1]. The present analyses show that an inverse relationship can be obtained also with random numbers for variables with distributions similar to those of the real concentrations for OA, AA, and sum of the remaining fatty acids. Thus, the inverse association between OA and AA apparently seems to have - at least partly - a non-biological explanation, thereby raising the question of whether our previous finding is just a type of spurious correlation. However, the finding that the correlation coefficient and scatterplot obtained with real values were superior to the ones found with random numbers suggests that the inverse association probably is mainly attributed to other mechanisms than Distribution Dependent Regulation. This conclusion seems to be supported by the absence of a significant %OA vs. %AA correlation when replacing real values with random ones in our previous studies in human and the rat sera [2, 3]. Nevertheless, the present analyses show that it is easy to be biased if relying on correlation coefficients only, and their levels of significance. Thus, also with random numbers we many times observed *p*-values less than 0.001 for the inverse “%OA vs. %AA” relationships, in spite of poor scatterplots. This finding strongly suggests that scatterplots should always be made when relating percentages of the same sum. Additionally, also a control with random numbers would seem justified. Anyhow, the present analyses suggest that our previous finding of an inverse relationship between percentages of OA and AA is not solely attributed to a Distribution Dependent Regulation, as a kind of correlation bias.

### Comment on the correlation between percentages of fatty acids

It is not surprising that percentages of fatty acids may be correlated, since they are all computed from the same sum. Indeed, as early as in 1897 Karl Pearson [29] reported that there will be a spurious correlation between two indexes with the same denominator, even if the variables used to produce the indexes are selected at random with no correlation between them. This general rule certainly applies to the present findings. However, our results show that a significant correlation between percentages of the same sum is not always obtained, and add that the *distribution* of the variables is essential for the outcome.

The histograms we made showed great variations in the *inte*r-individual distributions of particular fatty acids. It seems reasonable to suggest that there are *intra*-individual variations as well, for example related to time, diet, and environment in general. Possibly, the magnitudes of the *inter*- and *intra-*individual variations may not be very different. Thus, the Distribution Dependent Regulation might be encountered both between and within subjects.

Conceivably, percentages of two fatty acids can be inversely related since an increase in the percentage of one particular fatty acid must be accompanied by a reduced percentage of one or more of the remaining ones. However, percentages of two fatty acids may both be reduced- when the percentage of the remaining fatty acids increases. In this case the percentages of the two former ones may be positively related. While it is easy to conceive these general considerations, it is not so simple to predict whether percentages of two particular fatty acids (among several others) will be negatively or positively associated. As mentioned previously, we may try to predict the outcome by considering the equation %OA = − %AA + (100 – %R), but the exact outcome is hard to find without doing a computer analysis. Indeed, the distributions of OA and AA in breast muscle lipids of chickens are such that they do not clearly favor either a negative or a positive relationship between their relative amounts. This finding serve to explain why even small changes in distributions made appreciable changes in the “%OA” vs. “%AA” relationship. Nevertheless, we were surprised to see the strength of the distribution dependent correlations; the effect was as powerful as to make an inverse association become positive by small alterations in distributions.

The primary aim of the present analyses was to elucidate to what extent the correlation between percentages of OA and AA might have a non-biological explanation. Our random number analyses strongly suggest that this is partly the case. However, the ranges of the numbers that we used were not truly random since they were generated within the real concentration distributions of OA, AA, and R (the remaining fatty acids). This restriction imposed upon our random numbers made us wonder whether the negative correlation found between %OA and %AA could depend on the particular interval from which the random numbers were picked. We hypothesized that, if distributions were essential, then we would expect that changes in the distribution should disturb the relationship between %OA and %AA, and so was indeed observed in our analyses. It would accordingly appear that % OA and %AA must – at least in part -be inversely associated as a consequence of their particular distributions. We are currently investigating whether correlations between percentages other fatty acids may at least partly be explained by their distribution pattern. Indeed, the positive association between percentages of AA and EPA in breast muscle lipids of chickens seems to be completely explained by the particular concentration distribution of these fatty acids, possibly suggesting a Distribution Dependent Regulation [26, 27].

Our current examples with random numbers suggest that the concentration distribution per se of fatty acids may govern whether their relative amounts are positively or negatively associated.

Our previous [27, 28] and present findings lead to the intriguing question of whether evolution might have chosen particular concentration ranges for some fatty acids, to ensure that their relative amounts must be positively associated whereas percentages of others could be negatively correlated. From the present results we are not able to conclude on this issue. However, we find it hard to accept that a small change in the physiological distribution of OA (for example from 1 to 7 to 2–7 g/kg), making the negative %OA vs. %AA association become non-significant, really is of biological significance. On the other hand, if accepting that a negative (positive) relationship between percentages of fatty acids has any biological interest, it follows that also the *Distribution Dependent Regulation* could be of physiological interest. Further studies are required to elucidate whether this phenomenon should be considered as a correlation bias only or as a possible physiological regulatory mechanism.

## Conclusions

The present analyses suggest that the distribution per se of fatty acid concentrations may have the power to govern whether an association between their relative abundances will be positive or negative, and show that even small variations in the concentration distribution may alter such relationships. We do not know whether a disturbance in this type of regulation could be related to the risk of AA associated conditions and diseases. The present results support our previous observation that percentages of oleic acid and arachidonic acid are inversely related, and add that *Distribution Dependent Correlations* might partly govern the inverse relationship.

## Abbreviations

**OA**: is used for oleic acid (18,1 n9), and also for **a random number variable** with distribution like that of oleic acid; in the figure texts upper case letters (RANDOM) indicate that random numbers were used to produce the figure.
**AA**: is used for arachidonic acid (20,4 n6), and also for **a random number variable** with distribution like that of arachidonic acid; in the figure texts upper case letters (RANDOM) indicate that random numbers were used to produce the figure.
